# Mesenchymal Stem Cells and Their Small Extracellular Vesicles as Crucial Immunological Efficacy for Hepatic Diseases

**DOI:** 10.3389/fimmu.2022.880523

**Published:** 2022-05-06

**Authors:** Yuting Tang, Peipei Wu, Linli Li, Wenrong Xu, Jiajia Jiang

**Affiliations:** ^1^ Aoyang Institute of Cancer, Affiliated Aoyang Hospital of Jiangsu University, Suzhou, China; ^2^ Zhenjiang Key Laboratory of High Technology Research on Exosome Foundation and Transformation Application, School of Medicine, Jiangsu University, Zhenjiang, China

**Keywords:** hepatic diseases, mesenchymal stem cells, small extracellular vesicles, immunomodulatory effects, tissue regeneration

## Abstract

Mesenchymal stem cell small extracellular vesicles (MSC-sEVs) are a priority for researchers because of their role in tissue regeneration. sEVs act as paracrine factors and carry various cargos, revealing the state of the parent cells and contributing to cell–cell communication during both physiological and pathological circumstances. Hepatic diseases are mainly characterized by inflammatory cell infiltration and hepatocyte necrosis and fibrosis, bringing the focus onto immune regulation and other regulatory mechanisms of MSCs/MSC-sEVs. Increasing evidence suggests that MSCs and their sEVs protect against acute and chronic liver injury by inducing macrophages (MΦ) to transform into the M2 subtype, accelerating regulatory T/B (Treg/Breg) cell activation and promoting immunosuppression. MSCs/MSC-sEVs also prevent the proliferation and differentiation of T cells, B cells, dendritic cells (DCs), and natural killer (NK) cells. This review summarizes the potential roles for MSCs/MSC-sEVs, including immunomodulation and tissue regeneration, in various liver diseases. There is also a specific focus on the use of MSC-sEVs for targeted drug delivery to treat hepatitis.

## Introduction

Mesenchymal stem cells (MSCs) have gained attention for their potential in treating various diseases, advancing from animal experiments to clinical trials (1), and MSC biosafety has been widely indicated. We defined the renovation of MSCs in hepatic diseases over a decade ago using an in-house isolation culture system (2–7). MSCs differentiate into hepatocyte-like cells (8), informing the transition from liver to hepatocyte transplantation to improve liver function. The capacity of MSCs to reverse mouse hepatic injury (2) and *Schistosoma japonicum*-induced mouse liver injury was also shown (7). ERK1/2 phosphorylation was defined as an essential factor for differentiation (3), and concurrent overexpression of miR-106a, miR-574-3p, and miR-451 was shown to induce human umbilical cord-derived MSC (hucMSC) differentiation into functionally mature hepatocytes (9).

**Figure 1:**
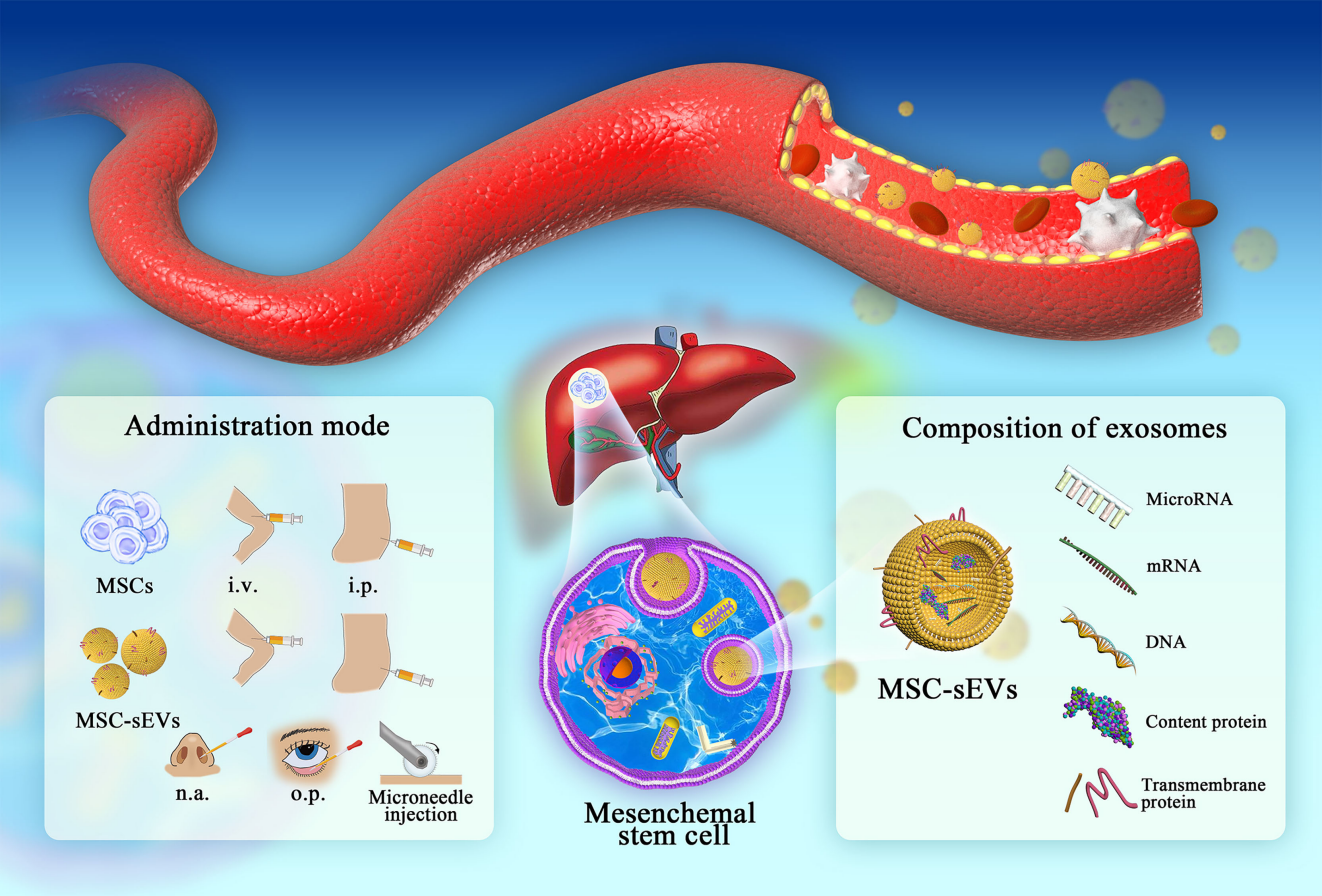
The administration of MSCs/MEC-sEVs and detailed composition of MSC-sEVs (i.v., intravenous injection; i.p., intraperitoneal injection; n.a., nasal drip; o.p., eye drop.). In liver diseases, MSC colonization and sEV circulation play important roles in damage repair. Research on the composition and administration of sEVs provides a novel chance for follow-up clinical promotion.

MSCs repair liver damage through both differentiation and exocrine regulation (10). Exosomes are nanosized (30–150 nm) membrane vesicles with a lipid bilayer that contains protein, DNA, RNA, and lipid molecules. The International Society for Extracellular Vesicles proposed membrane vesicles of 30-200 nm collectively referred to as small extracellular vesicles (sEVs) (11). These vesicles are classified as an exocrine form of MSCs that regenerate damaged tissues and organs, being considered as a novel type of intercellular communication. Disease occurrence and progression can be mapped by the transmission of sEVs. This paves the way for the use of sEVs as diagnostic markers and therapeutic targets, especially given that they offer more flexible modes of administration than MSCs (**Figure 1**). In this context, we have shown that MSC-sEVs can suppress hepatic oxidant injury and liver fibrosis by mediating immune responses (4–6).

Although MSCs/MSC-sEVs play an important role in regulating inflammatory diseases, the potential mechanisms by which they regulate immune cells and reduce immune-mediated injury have not been fully clarified. In this review, we will discuss the prominent immunomodulatory mechanisms involved in MSCs/MSC-sEVs’ therapeutic potential, and we will unveil a reasonable modification to MSCs/MSC-sEVs for treating diverse hepatic injury, including acute and chronic hepatitis, metabolic liver diseases, self-development and complications of liver cirrhosis, and hepatocellular carcinoma (**Table 1**).

**Table 1:** Studies testing the immunomodulatory effects of MSCs/MSC-sEVs on liver disease.

Diseases	Origin of MSCs/MSC-sEVs		Species	Dose	Application	Ref
ALF		HucMSC-sEVs	Male C57BL/6	—	Overexpression of miR-455-3p attenuates MΦ infiltration and local liver damage and reduces the serum levels of inflammatory factors	(12)
		HADSCs	Balb/c mice	4 × 10^5^ *via* tail vein	Lipid-conjugated heparin coat results in enhanced delivery, longer retention, faster decrease in AST and ALT, and alleviated inflammatory response	(13)
		HucMSCs	Balb/c mice	5 × 10^5^ *via* tail vein	Engrafts in the injured liver and repairs damaged tissue	(14)
		HucMSCs	Male mice	1 × 10^6^ intravenously	Inhibits inflammatory cytokine and chemokine levels and reduces immune cell infiltration into the liver tissue, also attenuates hepatocyte apoptosis	(15)
		Rat BMSCs	Male SD rats	1 × 10^7^/kg *via* tail vein	IL-1β-pretreatment enhances efficacy on survival rate, liver function, and liver necrosis suppression with more MSCs migrated to the damaged liver	(16)
		HucbMSCs	Male SD rats	1 × 10^7^ in *via* tail vein	VEGF165 overexpression enhances the multipotency of MSCs in stronger effectiveness, homing, and colonization	(17)
		BMSCs	SD rats	2.4 ml of CM for 3 consecutive days	Has higher cell viability and total protein synthesis *in vitro*, prevents the release of liver injury biomarkers, and promotes the recovery of the liver structure *in vivo*	(18)
		BMSCs	Male C57BL/6J	2 × 10^6^ *via* tail vein	MSC-derived PGE2 inhibits inflammatory activation of MΦ, promotes inflammatory resolution, and limits injury	(19)
		EMSCs	Syngeneic male SD rats	CM of 5 × 10^6^ inject intraperitoneally	Magnetic graphene oxide enhances the effect on reducing necrosis, inflammation, AST, ALT, and ALP, as well as the VEGF and MMP-9	(20, 21)
Hepatitis B		HBMSCs	Balb/c mice	1 × 10^6^ *via* tail vein	Influences innate immunity and limits immune-mediated liver injury by suppressing NK-cell activity	(22)
		HBMSCs	Human	1–10 × 10^5^/kg once a week for 4 weeks	Significantly increases the 24-week survival rate by improving liver function and decreasing the incidence of severe infections safely and conveniently	(23)
		HucMSCs	Human	1 × 10^8^ *via* infusion pump	Further improves the hepatic function and survival	(24)
		HucMSCs	Human	10^5^/kg intravenously	No significant advance in the short-term prognosis with further evaluated needed in the long-term efficacy	(25)
Hepatitis C		Mice BMSCs	H-2d mice	5 × 10^5^ *via* tail vein	Exhibits promising adjuvant property	(26)
		Autologous hBMSCs	Human	Fasting for intravenous infusion 1 × 10^6^/kg	Supportive in the treatment of end-stage liver disease, with satisfactory tolerability and beneficial effects on liver synthetic functions and hepatic fibrosis.	(27)
		Mice BMSCs	H-2d mice	5 × 10^5^ *via* tail vein	Enhances immune response	(28)
NASH		HucMSCs	C57BL/6 mice	200 ml CM/3 days for 2 months	Improves insulin resistance, amends pathological structure, enhances total antioxidant capacity and mitochondrial function, and reduces inflammation and apoptosis	(29)
		HADSCs	C57BL/6J mice	—	Reduces systemic inflammation and fat accumulation in the liver, increases browning of white adipose tissue depots, and improves glucose tolerance	(30)
		HADSCs and HADSC-sEVs	*Mc4r*-KO C57BL/6J mice	1 × 10^6^ cells; 1.0, 2.5, or 5.0 mg	Decreases serum ALT levels and inflammatory markers, improves fibrosis, and increases anti-inflammatory MΦ	(31)
		Rat BMSCs	Male SD rats	2 × 10^6^ *via* tail vein	Ameliorates liver lipotoxicity and metabolic disturbance	(32)
AIH		Mouse ADSCs	C57BL/6J mice	—	IL-35 gene modification manages MSCs to better migrate to injured liver tissues, narrows the necrosis areas of injured livers, and prevents the hepatocyte apoptosis	(33)
		BMSC-sEVs	C57BL/6 mice	2 µg/g	Attenuates inflammatory responses and inflammatory cytokine release in both the liver and MΦ	(34)
		BMSC-sEVs	Wild-type male C57BL/6 mice	20 μg/ml	Reverses injury in mice and hepatocytes, and downregulates the expressions of cytokines, NLRP3, and caspase-1	(35)
Liver fibrosis		ADSCs	C57BL/6 mice	400 μl CM	Decreases hepatic enzymes and collagen deposition	(36)
		Mice BMSCs	C57BL/6 mice	1 × 10^6^	Erythropoietin-overexpressed MSCs significantly alleviate liver fibrosis	(37)
		HucMSCs	C57BL/6 mice	5 × 10^5^ *via* tail vein	Ameliorates liver fibrosis, attenuates collagen deposition, and improves liver function	(38)
		ADSC-sEVs	C57BL/6J mice	0.4 μg/μl, 100 μl	Inhibits collagen volume fraction and reduces inflammatory factor levels and hepatic injury-associated indicators.	(39)
		HBMSC-sEVs	SD rats	250 mg	Alleviates liver fibrosis with a reduction in collagen accumulation, enhancement in liver functionality, inhibition of inflammation, and raise in hepatocyte regeneration	(40)
		Rat BMSCs	Albino rats	3 × 10^6^	Reverses the deterioration of liver function	(41)
		HTMSC-sEVs	C57BL/6 mice	150 mg/mouse	Attenuates HSC activation and liver fibrosis through miR-486 in sEVs	(42)
Liver cirrhosis		HBMSCs and hADSCs	C57BL/6 mice	1 × 10^6^	Attenuates liver damage, improves liver function, and regresses liver fibrosis	(43)
		HucMSCs	Human	3 times of 0.5× 10^6^/kg at 4-week intervals	Markedly improves liver function with a significant higher overall survival rate	(44)
		Rat BMSCs	Wistar rats	3–5 × 10^6^ *via* surgical incision	Mitigates liver cirrhosis	(45)
		Autologous hBMSCs	Human	5 × 10^7^ once/twice	Safely improves histologic fibrosis and liver function in patients with alcoholic cirrhosis	(46)
Liver GvHD		HucMSCs	Human	1 × 10^6^/kg body weight	Decreases ALT level and remains lower; increases Treg/Th17; increases transforming growth factor β1 and prostaglandin E2	(47)
		HBMSCs	Human	1.5–3 × 10^6^/kg on postoperative day 3	Long-term results of feasibility, safety, and tolerability of MSC infusion	(48)
		HBMSCs	Human	1–2 × 10^6^/kg	Induces mild positive changes in immunoregulatory T and NK cells	(49)
		HucMSCs	Human	1 × 10^6^/kg	Decreases the need for interventional therapies and improves the graft survival rates	(50)
		HucMSCs	Human	1 × 10^6^/kg	Decreases the incidence of acute rejection, rates of biliary complications, and infection	(51)
HCC		Rat BMSCs	Rat	1 × 10^6^/ml PBS	Melatonin pretreatment yields MSCs a better ameliorative effect	(52)
		HBMSC-sEVs	Nude mice	—	Reduces proliferation, migration, invasion, and self-renewal abilities of HCC cell lines	(53)
		HBMSCs	Nude mice	Intravenously inject 1 × 10^6^ cells at days 9 and 13	Modification of the virus’ fiber domain in MSCs results in a high level of virion accumulation in HCC and potent tumor growth inhibition	(54)
		HAMSC-sEVs	Male Balb/c nude mice	10 mg/kg once per week	miR-199a-modified MSCs effectively deliver miR-199ay to HCC, inhibit the mTOR pathway, and improve HCC chemotherapy	(55)
		HucMSCs	Male Balb/c nude mice	A mixture of hepatoma cells and MSCs equally	Inhibits the growth of liver cancer cells	(56)
		HBMSCs	Female CD1 nu/nu mice	0.5 × 10^6^ CMV-NIS-MSCs	MSCs show temperature-dependent migration	(57)
		HPMSCs	Male athymic nude mice	5 × 10^5^ *via* tail veins or into the tumor margin	MSCs combined with chemotherapy yields favorably result with higher tumor necrosis and greater proportion of apoptotic-positive cells	(58)
		HBMSC-sEVs	Balb/c mice	2 mg/kg	Delivery of anticancer drug norcantharidin induces cell-cycle arrest, reduces tumor cell proliferation, increases apoptosis, and exerts obvious *in vivo* antitumor effects	(59)

MSCs, mesenchymal stem cells; sEVs, small extracellular vesicles; HucMSCs, human umbilical cord-derived MSCs; ADSCs, adipose-derived MSCs; BMSCs, bone marrow-derived MSCs; PMSCs, placenta-derived MSCs; HTMSCs, human tonsil-derived MSCs; AMSCs, amnion-derived MSCs; HucbMSCs, human umbilical cord blood-derived MSCs; EMSCs, embryonic stem cell-derived MSCs; CM, conditioned medium; ALF, acute liver failure; AH, alcoholic hepatitis; NASH, non-alcoholic steatohepatitis; AIH, autoimmune hepatitis; HSC, hepatic stellate cells; GvHD, Graft versus host diseases; HCC, hepatocellular carcinoma; SD, Sprague-Dawley; Mc4r-KO, melanocortin type-4 receptor knockout; H-2d mice, mice of the DBA/2J (H-2d) line; alga-PEG, alginate-polyethylene glycol; CMV, Cytomegalovirus; NIS, theragnostic sodium iodide symporter.

## MSCs/MSC-sEVs as Neotype Immunosuppressants

**Figure 2:**
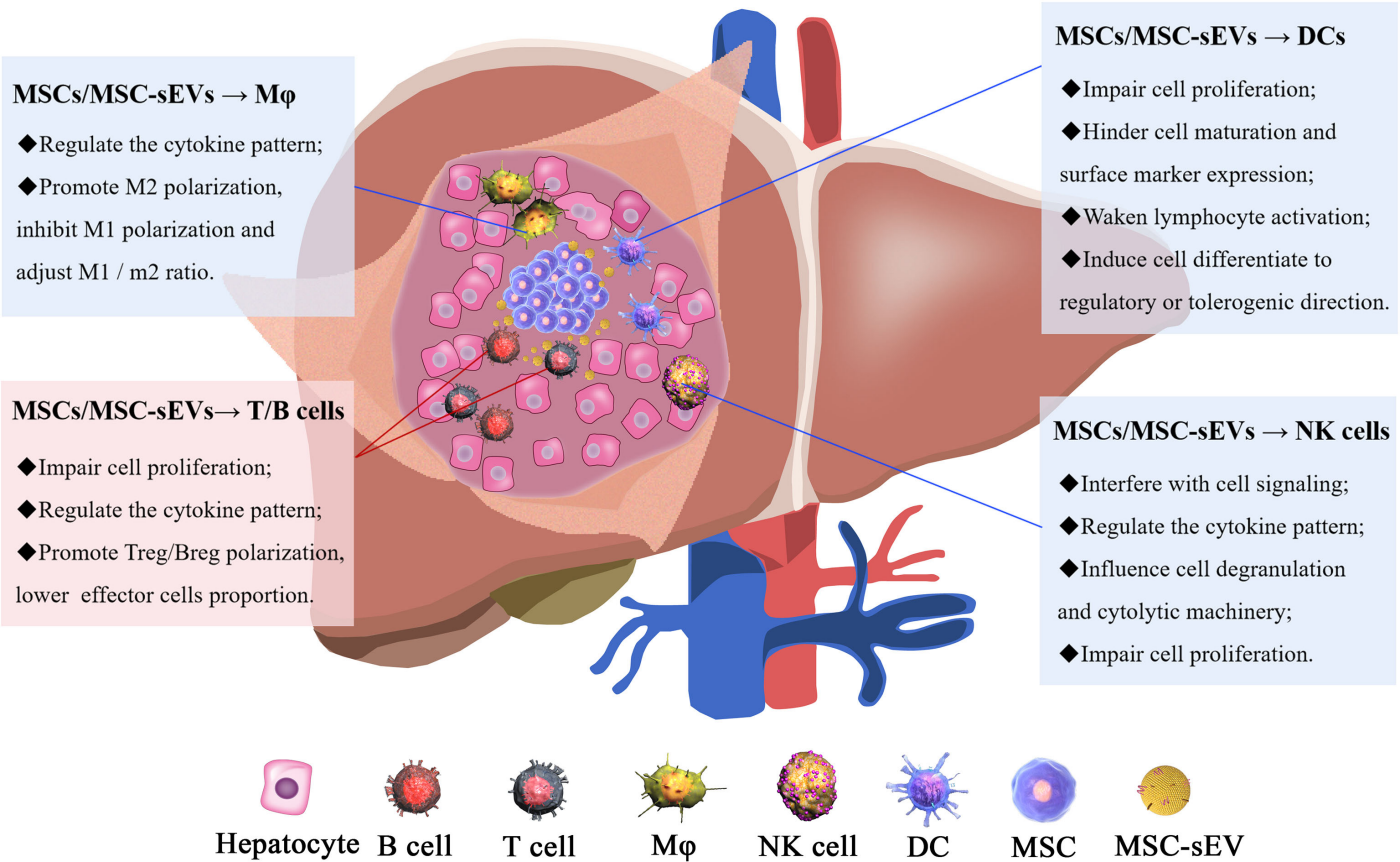
The mechanisms of MSC/MSC-sEV modulation of immune cells. The immunomodulatory effect of MSCs/MSC-sEVs is reflected in their ability to inhibit immune cell proliferation and function and induce anti-inflammatory cell activation.

To combat liver injury, tissues initiate innate and adaptive immune responses by inducing immune cell activation. An imbalance between liver injury and the immune response results in inflammation and further damage; thus, immunosuppression is necessary for hepatocytic regeneration and damage repair (60). MSC/MSC-sEV treatment reduces the secretion of the pro-inflammatory cytokines, IL-6, IL-1β, IL-12, IFN-α, IL-2, and IL-4 and increases the production of the anti-inflammatory cytokines, TGF-β, and IL-10 (61–63), leading to immunosuppression (**Figure 2**).

### Innate Immune Responses

MSCs and their sEVs effectively regulate innate immune cells such as DCs, NK cells, and MΦ. DC maturation and proliferation can be suppressed by MSCs/MSC-sEVs (64). Zhao et al. (65) discovered that DCs suppress inflammation through the CD200/CD200R signaling pathway. MiR-21-5p is enriched in MSC-sEVs, targeting degradation of CCR7 on DCs and modulating cell function (63). Yuan et al. (64) demonstrated that the IFN-γ/FLT3L-FLT3/CD1c^+^ DC axis plays a role in upregulating tolerogenic CD1c^+^ DCs and suppressing inflammation in response to MSC treatment. Regulatory DCs are characterized by low costimulatory molecule expression and may be synergistically stimulated by MSCs through Notch (66) and TGF-β signaling (67). In brief, insufficient maturation and reduced expression of DC maturation and activation markers (61) may impair antigen uptake and decrease lymphocyte proliferation (63).

MSCs/MSC-sEVs exert an immunomodulatory effect on NK cells by interfering with cell signal transmission, regulating cytokine secretion, preventing degranulation and cytolysis, and reducing proliferation (68). MSCs strongly decrease NK cell-induced IFN-γ production by secreting activin-A (69). While both MSC-derived conditioned medium (CM) and NK-MSC-cocultured CM prevent NK cell degranulation, the effect is more dramatic for cocultured CM. This finding suggests that the suppressing factor is increased when MSCs contact NK cells (70). In addition, MSCs that have been pre-activated by inflammatory cytokines secrete prostaglandin E2 to promote NK-cell inhibition and reduce the expression of the activation marker, CD69, the cytotoxic ligand, TRAIL, and the chemokine receptor, CXCR3, on the NK cell surface (71).

MSCs/MSC-sEVs promote MΦ polarization toward the M2-like phenotype and regulate the release of inflammatory factors. MSCs also inhibit MΦ polarization toward the CD86^+^ M1 phenotype through reduced production of NO and regulated production of pro-inflammatory factors like TNF-α, IL-1β, IL-6, IL-8, and NOS-2 (72). A significant decline in the M1/M2 MΦ ratio is observed following MSC or MSC-sEV treatment (73). Mechanistically, TGF-β secreted by MSCs facilitates the polarization of CD163^+^ M2 MΦ through the Akt/FoxO1 pathway (74), while MSC-sEVs deliver IL-10 to Kupffer cells and induce the expression of PTPN22, shifting Kupffer cells to an anti-inflammatory phenotype and mitigating liver inflammation (75). Enrichment of miRNA in MSC-sEVs negatively regulates the inflammatory environment by miR-223-3p targeting stathmin 1 (76), miR-21 targeting programmed cell death 4 (77), and miR-124-3p targeting nucleus signaling 1 (78).

### Adaptive Immune Responses

T and B cells are directly modulated by MSCs/MSC-sEVs and are also affected by MSC-induced immunosuppression of innate immune cells (79). MSCs/MSC-sEVs limit the proliferation and activation of CD4^+^ T (80), CD8^+^ T (81), γδ T (82), and B cells (83). MSC-induced chitinase-3-like protein 1 inhibits STAT1/3 signaling in T cells by upregulating peroxisome proliferator-activated receptor δ (84). Naïve MSCs have a greater impact on T cells (79), while IFN-γ-primed MSCs strongly inhibit B cells (79, 85). Lee et al. (86) showed that phorbol myristate acetate primes MSCs to attract B cells in a CXCL10-dependent manner and induces apoptosis and concomitantly inhibits IgG production in a PD-L1-dependent manner. IgM also significantly decreases because of altered bio-function genes involved in cell-to-cell signaling and interaction and cellular movement (83).

MSCs/MSC-sEVs also induce Treg/Breg-cell differentiation. Promoting Treg differentiation (87) reverses the imbalance of T effector and Treg cells (88). MSC-sEVs induce differentiation through the miR-1246/Nfat5 axis (89) and the mTOR-mediated axis (90) and the secretion of hepatocyte growth factor by MSCs (91). The transfer of mitochondria from MSCs to T cells induces Treg-cell differentiation through an increase in Treg cell differentiation-related gene FoxP3 expression (92, 93). The anti-inflammatory factors, TGF-β, PGE2, and IL-10, secreted by MSCs contribute to Breg-cell generation and prevent T effector-cell proliferation and inflammatory factor secretion (94, 95). The ability of MSCs/MSC-sEVs to modulate the phenotype, function, and homing of immune cells could inform new therapies for inflammatory and autoimmune diseases.

## Insights From Hepatic Diseases

The liver is an essential organ to maintain life activities, breaking down and creating nutrients and aiding energy metabolism (96). Hepatitis is divided into acute and chronic forms that can be caused by viruses, bacteria, parasites, chemical poisons, drugs, alcohol, or autoimmune factors. During chronic liver injury, collagen accumulation can lead to cirrhosis and eventually develop into hepatocellular cancer (97). Although vaccination and drug development have reduced the occurrence and progression of particular liver diseases, the prevalence and burden of these illnesses remain significant (97).

### Acute Liver Failure

Acute liver failure (ALF) is a life-threatening clinical syndrome characterized by sudden massive hepatocellular necrosis and coagulopathy (98). Both human adipose-derived MSCs (hADSCs) (13) and hucMSCs (14) are effective against ALF, relieving hepatotoxicity, promoting hepatocyte regeneration (15), and alleviating inflammation. PGE2 secreted by BMSCs blocks MΦ activation by inhibiting TGF-β-activated kinase 1 (TAK1) signaling and the NLRP3 inflammasome and induces M2 MΦ by activating STAT6 and rapamycin (mTOR) signaling (19). Lipid-conjugated heparin was designed to enhance the delivery and retention of MSCs for better engraftment in the injured liver and improve repair of damaged tissues (13). Importantly, pro-inflammatory factor pre-stimulation may improve the therapeutic capacity of MSCs. IL-1β pretreatment (16) and VEGF_165_ gene modification (17) enhance MSC multipotency and improve their effectiveness, homing, and colonization. The therapeutic factor, miR-455-3p, targets PI3K signaling, ameliorating ALF as an sEV cargo (12).

MSC-CM, which contains a high number of cytokines and paracrine factors secreted by MSCs, including MSC-sEVs, has been used as a therapeutic agent in some studies (18, 20). After demonstrating the reversal of ALF in mice that received MSC-CM treatment, Chen et al. (18) investigated the effects of CM derived from MSCs that were cocultured with hepatocytes and showed that it prevented the release of liver injury biomarkers and promoted the recovery of liver structure. The introduction of silica magnetic graphene oxide increased the ability of MSC-CM to reduce necrosis, inflammation, and liver biochemical levels (20, 21).

### Viral Hepatitis

Hepatitis pathogens entering the human body through routes like the skin and blood result in an infectious disease primarily caused by liver lesions. sEV-mediated infection is an essential route of viral transmission by carrying the structural protein and nucleic acid of the virus that is produced by infected cells (99–101). The release of sEVs provides pathogens with a mechanism for escaping the host immune system by impairing immune cell function (102). Blocking sEV release from infected cells prevents virus replication (101). Based on these findings, sEVs have been used as vectors for vaccine delivery (103). Masalova et al. (26) revealed that MSCs have adjuvant properties during DNA vaccination against hepatitis C. MSCs modified with five non-structural hepatitis C virus proteins resulted in a pronounced innate and adaptive immune response than DNA immunization, suggesting that MSCs may serve as an effective antiviral vaccine (28).

A recent study (22) assessed the interaction between NK cells and BMSCs during hepatitis B virus (HBV) infection. *In vivo* results showed that MSCs accumulate in the injured liver and minimize liver damage. However, limiting immune-mediated liver injury was at the cost of suppressing NK-cell activity and restricting innate immunity, which could promote HBV gene expression and replication. In a randomized controlled trial (23), patients with HBV-related acute-on-chronic liver failure received an allogeneic hBMSC infusion. While fever was more common for patients in the MSC group, serum total bilirubin levels were improved and end-stage liver disease scores were associated with less severe infection and a lower incidence of multiple-organ failure. Using the same injection method, Li et al. (24) showed significant improvement in liver function following hBMSC treatment while Xu et al. (25) found no short-term improvements. These differences may be explained by the different numbers of patients and MSC doses used in the two studies. A safe and effective case for autologous MSC infusion was reported by Salama et al. (27). The tolerability and beneficial effects on liver function and hepatic fibrosis are encouraging.

### Liver Injury Complicated by COVID-19

COVID-19, which continues to sweep the globe, is commonly associated with complicated liver injury (104) which is severe in some cases (105). Fang et al. (106, 107) found that prognosis is particularly poor for COVID-19 patients with chronic HBV-infection. Moon et al. (108) and Iavarone et al. (109) both found higher mortality among patients with cirrhosis who were subsequently infected with COVID-19. Considering the multiple-organ failure associated with this disease, MSC/MSC-sEVs may be a potential treatment (110). The liver injury caused by COVID-19 may not result from direct viral invasion, but rather the cytokine storm caused by infection (104, 111). MSC/MSC-sEV administration results in MΦ polarization from the M1 to the M2 reparative phenotype (73) and immune suppression that can attenuate the cytokine storm (112).

In clinical remission of critically ill COVID-19 patients, the triple administration of allogenic hucMSCs with a 3-day interval, together with daily thymosin α1 and antibiotic injection, alleviated inflammation after the regular antiviral therapy was found to be ineffective (113). A clinical trial conducted in China showed the safety and efficacy of intravenous MSC transplantation among patients with COVID-19 pneumonia, especially those in critically severe conditions (114). HucMSC treatment effectively reduces serum IL-6 levels in patients, which is considered a biologically relevant biomarker for COVID-19 disease progression (115, 116). Emerging clinical evidence indicates that MSC treatment is safe and has fewer side effects, suggesting that it could be an effective therapy for patients with severe COVID-19 (**Table 2**).

**Table 2:** Ongoing or recently completed Chinese clinical trials with MSCs/MSC-sEVs in COVID-19.

Clinical trial registration	Study title	Status	Phase	Patients	MSC type	Dose and infusion
NCT04252118	MSCs treatment for pneumonia patients infected with COVID-19	Recruiting	I	20	HucMSCs	Conventional treatment plus 3 times of 3.0 × 10^7^ MSCs intravenously at day 0, day 3, and day 6
NCT04339660	Clinical research of human MSCs in the treatment of COVID-19 pneumonia	Recruiting	I/II	30	HucMSCs	1 × 10^6^ MSCs/kg body weight suspended in 100 ml saline intravenously
NCT04346368	BMMSCs treatment for severe patients with coronavirus disease 2019 (COVID-19)	Not yet recruiting	I/II	20	HBMSCs	Conventional treatment plus 1 × 10^6^/kg body weight MSCs intravenously at day 1
NCT04273646	Study of hucMSCs in the treatment of severe COVID-19	Not yet recruiting	Not applicable	48	HucMSCs	4 times of 0.5 × 10^6^/kg body weight MSCs intravenously at day 1, day 3, day 5, and day 7
NCT04288102	Treatment with hucMSCs for severe corona virus disease 2019 (COVID-19)	Completed	II	45	HucMSCs	3 doses of 4.0 × 10^7^ MSCs intravenously at day 0, day 3, and day 6
NCT04276987	A pilot clinical study on inhalation of MSC sEVs treating severe novel coronavirus pneumonia	Completed	I	24	HADSC- sEVs	5 times aerosol inhalation of 2.0 × 10^8^ MSC-sEVs/3 ml at day 1, day 2, day 3, day 4, and day 5
NCT04371601	Safety and effectiveness of MSCs in the treatment of pneumonia of coronavirus disease 2019	Active, not recruiting	I	60	HucMSCs	Conventional treatment plus 1 × 10^6^/kg body weight MSCs once every 4 days for a total of 4 times intravenously within 3 days of first admission
NCT04269525	HucMSCs treatment for the 2019-novel coronavirus (nCOV) pneumonia	Recruiting	II	16	HucMSCs	3.3 × 10^7^ MSCs/50 ml/bag, 3 bags each time, 1 time each day, infused intravenously on the 1st, 3rd, 5th, and 7th days after enrollment

### Alcoholic Hepatitis and Non-Alcoholic Steatohepatitis

Total sEV numbers are increased in the plasma of patients with both alcoholic hepatitis (AH) and non-alcoholic steatohepatitis (NASH) (117). In a study of the protein expression profiles of sEVs enriched from the serum samples of 24 patients diagnosed with various fatty liver diseases, 61 proteins provided a confident differentiation, of which the most significantly upregulated proteins were α-2-macroglobulin for AH and apolipoprotein C3 for NASH (118). Alcohol-treated hepatocytes release sEVs with liver-specific miR-122 (119) and CD40L (120), both of which contribute to MΦ activation. Curtis et al. (121) assessed the capacity of BMSCs to relieve liver and lung inflammation in intoxicated and burned mice, suggesting that MSCs could be a novel therapy for restoring the homeostasis of multiple-organ systems in intoxicated burn patients.

NASH is closely related to obesity, insulin resistance, type 2 diabetes, and hyperlipidemia. HucMSC-sEVs alleviate type 2 diabetes by reversing peripheral insulin resistance and relieving β−cell destruction in both the liver and muscle (122), which is consistent with findings by Yang et al. (29) and Domingues et al. (30). In a streptozotocin and high-fat diet (HFD)-induced T2DM mouse model, hucMSC-CM was reported to reverse liver dysfunction, enhance liver total antioxidant capacity and mitochondrial function, and decrease inflammation (29). ADSC-sEVs facilitate metabolic homeostasis in obese mice by polarizing MΦ to M2 and beiging in white adipose tissue, promoting ADSC proliferation and establishing a cross-talk that facilitates immune and metabolic homeostasis (123). In a preestablished *Mc4r*-KO NASH mouse model, hADSCs and their sEVs are both able to adjust the inflammatory environment and enhance liver function (31). BMSCs reproduce the corresponding therapeutic effect in a rat model, protecting hepatocytes from lipotoxicity by regulating endoplasmic reticulum stress and calcium homeostasis (32).

### Autoimmune Hepatitis

Autoimmune hepatitis (AIH) is a chronic liver disease characterized by positive autoantibodies (124) and featured with lymphocyte infiltration. MSC treatment can partially alleviate AIH (33). A recent study transfected IL-35, a cytokine involved in regulating Treg cells and essential in autoimmune diseases, into ADSCs to strengthen the protective effect against AIH (33). IL-35-ADSCs prevented hepatocyte apoptosis by reducing FASL expression and IFN-γ secretion by liver mononuclear cells, significantly reducing the necrotic area in injured liver. Both BMSC-sEVs and BMSC-sEVs^miR-223(+)^ significantly reversed AIH as shown by serum testing, liver histology, inflammation, and cell death, while BMSC-sEVs^miR-223(-)^ counteracted the therapeutic effect of sEVs, indicating a possible mechanism related to miR-223 enrichment in sEVs (35). A follow-up study (34) specific to miR-223-3p showed a reduction in the release of inflammatory cytokines by MΦ.

### Liver Fibrosis and Cirrhosis

Liver repair involves the process of fibrosis to protect tissue integrity when the injury exceeds the regeneration ability of parenchymal cells, resulting in excessive deposition of the extracellular matrix, activation of hepatic stellate cells (HSCs), and a decrease in parenchymal cells (125, 126). HucMSCs ameliorate HSC activation and liver fibrosis by upregulating miR-455-3p through suppression of p21-activated kinase-2 (38). Reversal of liver function and histological damage was observed after BMSC injection (41). ADSC-CM reduced hepatic enzyme levels and collagen deposition (36), and ADSC-sEVs inhibited HSC proliferation by transferring miR-150-5p to reduce CXCL1 expression (39). BMSC-sEVs regulate Wnt/β-catenin signaling (40) while tonsil-derived MSC-sEVs suppress hedgehog signaling (42), renewing hepatocyte and liver function and controlling inflammation. circDIDO1 originates from BMSC-sEVs sponge for miR-141-3p and further suppresses HSC proliferation by blocking PTEN/AKT signaling (127). Erythropoietin was used as a target factor in a study by Wang et al. (37), promoting MSC cell viability and migration by activating PI3K/AKT and ERK1/2 signaling pathways and boosting anti-fibrotic efficacy.

When there is persistent liver injury, fibrosis develops into cirrhosis. Effective cirrhosis treatment suppresses HSC activation and fibrogenesis and induces fibrinolysis and the production of anti-inflammatory factors (128). MSCs strongly induce anti-inflammatory MΦ and, in combination with the medicine Juzentaihoto, activate NK cells and Treg cells to further attenuate liver damage and reverse liver fibrosis (43). Smad7-expressing MSCs treated liver fibrosis using the TGF-β1/Smad signaling (45). In clinical trials, hucMSCs can promote long-term survival with favorable liver function (44) while autologous hBMSCs suppress histologic fibrosis in patients with alcoholic cirrhosis in a dose-dependent manner for fibrosis quantification and Child–Pugh scores (46).

### Complications After Liver Transplantation

Liver transplantation is typically the last resort for end-stage liver disease (129). Ischemia–reperfusion injury (IRI) and biliary complications are postoperative responses to liver transplantation (130). HucMSC-sEVs modulate CD154 expression on intrahepatic CD4^+^ T cells and interfere with the activation of subsequent effector cells (131), protecting against IRI-induced hepatic apoptosis both *in vivo* and *in vitro* (132). In a recent study, 12 patients with biliary tract lesions were recruited into the MSC group and after six doses of hucMSCs, there was a reduction in the biochemical indexes of their liver function with no short- or long-term adverse events, except only one self-limiting fever (50).

While the incidence of liver graft versus host disease (GvHD) is relatively rare following liver transplantation, the conflict between the abundance of graft liver lymphocytes and the recipient’s main histocompatibility antigen means that GvHD is often lethal. The use of MSCs to inhibit GvHD has been moved into the preclinical phase (133). *In vivo* fluorescence tracked the biodistribution of hucMSCs labeled with semiconducting nanocrystals after tail-vein injection and confirmed their regeneration potential (134). After a single injection of hucMSCs to liver allograft recipients that had experienced acute rejection, ALT decreased markedly and remained low through the 12-week follow-up period alongside a significant increase in the Treg/Th17 cell ratio (47). A 5-year follow-up (48) verified the long-term feasibility, safety, and tolerability of MSC infusion. There is likely more potential for MSC infusion as a prophylactic before transplantation rather than a regulator after transplantation (133). In a randomized open-label phase Ib/IIa clinical trial, a single pre-transplant intravenous infusion of third-party BMSCs induced mild positive changes in Treg and NK cells in the peripheral blood with no complications indicated at the 1-year follow-up (49). As a result of the low sample size, this experiment did not obtain statistical significance. However, the slight increase in the proportion of Treg cells along with the anti-donor CD8^+^ T-cell hypo-responsiveness suggests that a pro-tolerogenic environment developed in MSC transplant recipients could eventually result in immune tolerance. The *ex vivo* delivery of active MSCs to liver grafts was proposed and expected for regeneration and immune regulation following transplantation (135).

To address the limitation of ABO-compatible liver transplantation, clinicians began the use of ABO-incompatible grafts as an alternative option, which may result in aggravation of the postoperative immune response (136). To overcome this, an anti-CD20 monoclonal antibody, rituximab, was introduced; however, severe adverse effects threaten the survival of transplant recipients (137). A randomized, open-label clinical trial replaced rituximab with hucMSCs for immunosuppression following ABO-incompatible liver transplantation (51). There were no significant differences in 2-year graft and recipient survival, and there was an obvious decline in biliary complications and infection in the MSC group.

### Hepatocellular Carcinoma

Liver cancer is usually the final sequelae of chronic liver disease given the high incidence of genetic alterations that accompany it (138). Hepatocellular carcinoma (HCC) accounts for a large proportion of liver cancer and is a leading cause of cancer-related death (139). Bioluminescence imaging has been applied to track the efficacy of tumor suppression following hucMSC treatment, and a significant variation in bioluminescence signal intensity indicates a positive role for MSCs in tumorigenesis (56). MSC-sEVs regulate the proliferation, migration, invasion, angiogenesis-stimulating, and self-renewal abilities of HCC cancer stem cells (53). miR-199a enriches MSC-sEV-sensitized HCC cells to doxorubicin by blocking the mTOR pathway (55). Wang et al. (57) proposed that tumors may have a specific impact on MSCs. Indeed, studies show a modest and largely reversible impact on MSC phenotype and glucose metabolism and a permanent impact on their secretory phenotype and tumor-promoting properties (57).

Hajighasemlou et al. (138) combined sorafenib, one of the standard treatments for advanced hepatocellular carcinoma, with MSCs to address drug resistance and side effects (58). MSCs, possessing well-established tumor-homing properties, serve as oncolytic adenovirus delivery tools. Under normoxic and hypoxic conditions, MSC infected with a low viral dose can effectively lyse HCC cells without significant hepatocellular toxicity (54). The anticancer drug, norcantharidin, was loaded into BMSC-sEVs to promote a continuous and slow release of the drug (59). MSCs pretreated with melatonin accepted the domestication of the tumor microenvironment and maximized the therapeutic outcome (52). MSCs were more attracted to heat-treated tumors both *in vitro* and *in vivo*, and MSC therapy with regional hyperthermia significantly enhanced the therapeutic efficacy (57). Numerous experiments have assessed the utility of MSC therapy to promote injury regeneration and immune regulation around the injury.

## Advances and Perspectives

**Figure 3:**
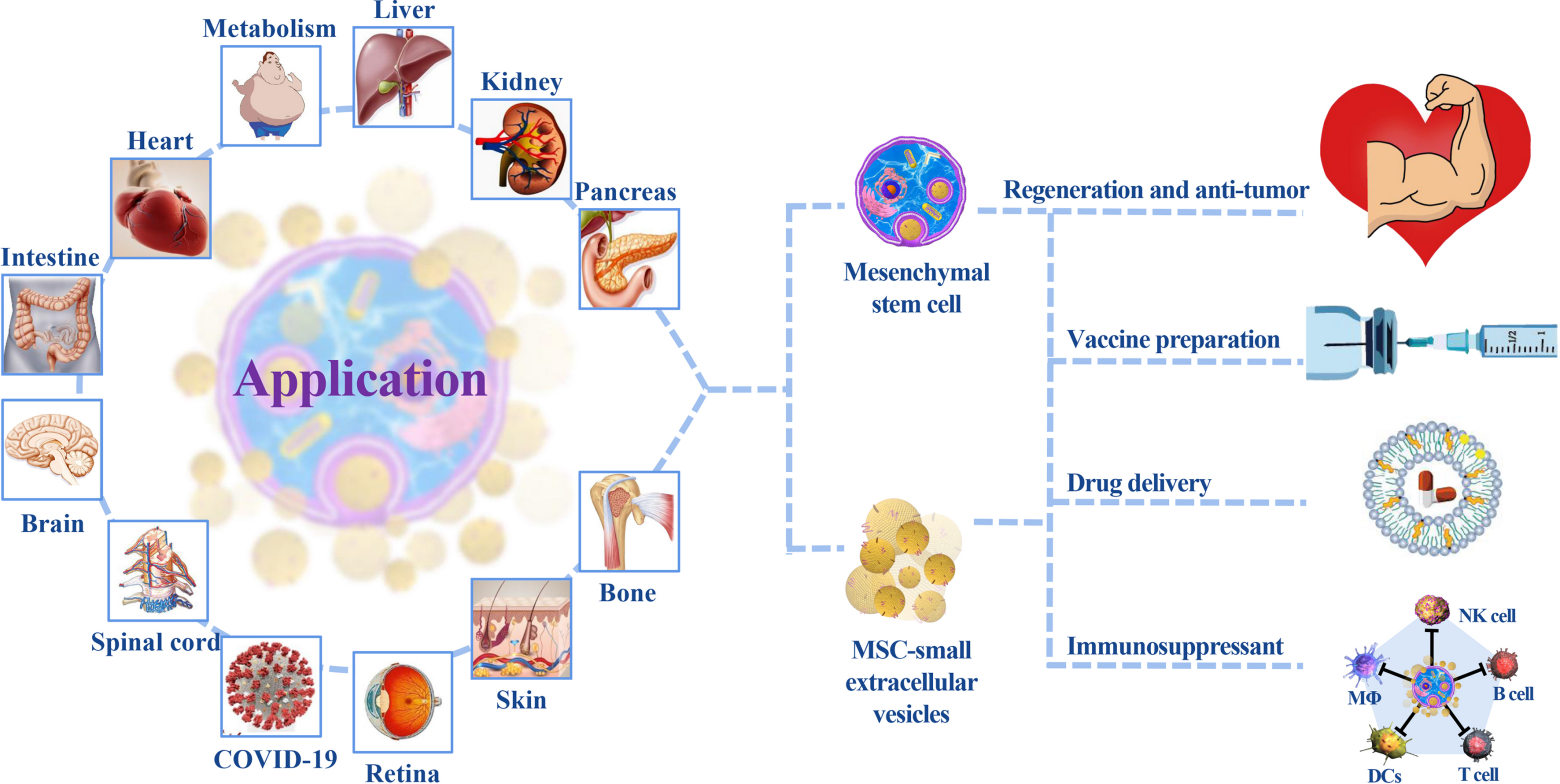
The application of MSCs/MSC-sEVs. MSC treatment primarily focuses on regenerative repair and autoimmune disease treatment. sEVs play a key role in cardiovascular, tumor, and nervous system diseases because of their diversified administration modes and ability to cross the blood–brain barrier. Their participation in multiple-organ repair provides insight into new medical technologies.

MSCs and their sEVs play an important role in tissue damage repair and tumor inhibition (140, 141). Their use in clinical practice is associated with improved prognoses (**Figure 3**) (44, 49, 142). Systemic regulation that relies on body fluid circulation and specific targeting of injured tissues make MSCs/MSC-sEVs relevant for the treatment of COVID-19 (143); a single administration is equivalent to a multidrug combination. While most research teams inject MSCs/MSC-sEVs in combination with antiviral therapy, this does not limit their importance (113–116, 144). However, a series of issues resulting in the slow progress of clinical treatment and development of MSCs/MSC-sEVs as an ideal treatment strategy remains to be addressed.

It is worth noting that the anticancer capability of MSCs was not unified across laboratories, because some experimental studies have indicated that MSCs/MSC-sEVs can promote cancer development (145, 146). This may be related to the methods used for cell extraction or sEV separation and purification. In addition, there are discrepancies in the therapeutic efficacy of MSCs/MSC-sEVs from different sources, species, and individuals (147). Thus, the donor selection of MSCs requires strict screening. For example, a pregnant woman’s physical status, age, history of metabolic, infectious, and familial genetic diseases, and fetal status need to be taken into account prior to hucMSC treatment.

In addition to the administered dose of MSCs/MSC-sEVs, a unified industry standard has not yet been established. In most experiments summarized in this paper, the MSC dose varies from 10^5^ to 10^8^ while the sEV dose ranges from μg to mg, indicating that most research teams agree that a particular concentration is essential, and increasing this could be as problematic as using too little. Partial experiments were conducted using dosage differences, and there was a certain correlation between the therapeutic effect and dosage (46). It was shown that hucMSC-sEVs accumulate in the liver after tail vein injection in normal mice. However, in mouse models of injury to other organs, sEVs target the injury site while still accumulating in the liver. Due to metabolic differences resulting from the route of administration (148) and phagocytosis (149) by mononuclear/MΦ, there are differences in the tendency of MSC-sEVs to migrate to different organs. The optimal dosage of MSCs/MSC-sEVs for different species and diseases remains a challenge for their use in clinical practice.

Research comparing outcomes to treatment with MSCs and their sEVs indicates that sEVs are superior (40). MSC and sEV treatment have their own merits. Cell therapy based on MSCs originated earlier than the sEV. Currently, clinical trials are mostly focused on MSC-based cell therapies. MSC treatment of liver disease contributes to both hepatocyte transformation and cytokine secretion, while sEV therapy is more one-sided. MSC infusion is often only required once, and MSC colonization at the injured site maintains a long-term effect, including the secretion of sEVs, as compared with the multiple infusions of sEVs required for metabolism. However, these benefits make it difficult to standardize the mechanism. Additionally, the safety of MSCs for aging and long-term tracking has not been confirmed. He et al. (150) proposed that dead MSCs may also play a role in immune regulation that is comparable to living cells, which impacts the original view of living cell therapy.

sEVs inherit the regenerative capacity of MSCs in the damaged environment and avoid the prognostic concerns caused by cell therapy. There is no unified standard for an optimal dose of sEV therapy. Cryopreservation (151) ensures long-term preservation, and convenient transportation avoids the wastage of biological activities, providing a strong guarantee for large-scale clinical promotion. The compilation of standards (T/CRHA 001-2021) in human MSC-sEVs by the Chinese Extracellular Vesicle Association has specified the preparation process of sEVs in detail, including storage and transportation, and is expected to standardize related scientific experiments. Naive sEVs can naturally be used as the targeted carriers of drugs or therapeutic molecules (152–154). Materials are applied externally to achieve a slow-release effect and consequently prolong the cycle time. Hydrogel, frequently used in medical materials, wrap up the sEVs and maintain their bioavailability so that they are not rapidly cleared under biodegradation. The sustained release is recorded for up to 1 month (155). The combination of modern drug therapy and material science greatly increases sEV acceptability and effectiveness.

Although the road from bench to bedside may be complex, MSCs/MSC-sEVs are a promising therapeutic agent for the treatment of tissue damage and tumors.

## Author Contributions

YT searched the literature and wrote the first draft of the manuscript. PW and LL critically revised the work. Corresponding authors WX and JJ are responsible for the guidance, revision, and fund support. All authors contributed to the article and approved the submitted version.

## Funding

This study was supported by grants from the National Natural Science Foundation of China (No. 81971757), the Jiangsu Province’s Major Project in Research and Development (BE2020680), Zhenjiang Key Laboratory of Exosome Foundation, and Transformation Application High-tech Research, China (SS2018003), the Clinical Major Disease Project of Suzhou (LCZX202019), the Technology Development Project of Suzhou (SKY2021018), and the Technology Project of Zhangjiagang (ZKCXY2106).

## Conflict of Interest

The authors declare that the research was conducted in the absence of any commercial or financial relationships that could be construed as a potential conflict of interest.

## Publisher’s Note

All claims expressed in this article are solely those of the authors and do not necessarily represent those of their affiliated organizations, or those of the publisher, the editors and the reviewers. Any product that may be evaluated in this article, or claim that may be made by its manufacturer, is not guaranteed or endorsed by the publisher.
